# Management of macroprolactinomas

**DOI:** 10.1186/s40842-015-0006-4

**Published:** 2015-07-20

**Authors:** Amit Tirosh, Ilan Shimon

**Affiliations:** 1 Institute of Endocrinology, Rabin Medical Center, Beilinson Campus, Petah Tiqva, 4941492 Israel; 2 Felsenstein Medical Research Center, Sackler School of Medicine, Tel Aviv University, Tel Aviv-Yafo, Israel

**Keywords:** Cabergoline, Dopamine agonist, Macroadenoma, Prolactinoma

## Abstract

Prolactin (PRL) secreting tumors are the most common functional neoplasms of the pituitary and are commonly subdivided into microprolactinomas (<10 mm) and macroprolactinomas (≥10 mm) according to their baseline diameter. Patients with prolactinoma present with symptoms evolving from hyperprolactinemia and with those caused by pressure of the expanding mass on surrounding tissues, including the optic chiasm and the cavernous sinuses. We hereby describe the possible complications of macroprolactinomas, including mass effects, hypopituitarism, CSF leak and apoplexy and discuss their relevant management.

In general, all patients harboring macroprolactinomas should be treated, the objectives being to achieve normal or near normal PRL levels, to reduce or stabilize adenoma size and to recover altered pituitary axes. Medical therapy with dopamine agonists (DA) is the preferred initial treatment for the vast majority of patients harboring prolactinomas. Pituitary surgery is indicated in patients who cannot tolerate or are resistant to therapy with DAs, patients that seek fertility and harbor adenomas that impinge on the optic chiasm, psychiatric patients with contraindication to DA treatment and patients presenting with pituitary apoplexy or a cerebrospinal fluid (CSF) leak. In addition, in this review, several patient populations with unique clinical characteristics will be discussed separately namely postmenopausal women, the elderly, children and patients with pituitary carcinoma.

## Introduction

Prolactin-secreting tumors are the most common functional neoplasms of the pituitary, accounting for 30-40 % of pituitary adenomas [1]. Prolactinomas are commonly subdivided into microprolactinomas (<10 mm) and macroprolactinomas (≥10 mm) according to their baseline diameter. Patients with prolactinoma can present with symptoms evolving from hyperprolactinemia which suppresses the gonadotroph axis and causes galactorrhea with amenorrhea or dysmenorrhea in women and erectile dysfunction and decreased libido in men. In addition, patients might describe symptoms caused by the pressure of the expanding mass on surrounding tissues, including the optic chiasm and the cavernous sinuses, resulting in visual disturbances; or in the compression of the normal pituitary gland, causing hypopituitarism [2, 3].

Several reviews have been published on diagnostic and therapeutic aspects of prolactinomas [4–8] and there are clinical guidelines for the management of prolactinomas [9, 10]. This review will focus on the practical management of patients with macroprolactinomas. The treatment objectives for patients harboring macroprolactinomas are to achieve normal or near normal prolactin (PRL) levels, to reduce or stabilize adenoma size and to recover altered pituitary axes. Below we describe the various methods used to achieve these goals, the efficacy of the different therapeutic tools and their possible side effects.

## Review

### Medical treatment

In general, all patients harboring macroprolactinomas should be treated. Specific indications include infertility, tumor mass effects, galactorrhea and hypogonadism [9]. Medical therapy with dopamine agonists (DA) is the preferred initial treatment for the vast majority of patients harboring prolactinomas [9–11].

There are several DA agents, including cabergoline (CAB), bromocriptine (BRC), and quinagolide. However, the agents in regular use are CAB and BRC [9]. Cabergoline is used more often due to several advantages over BRC. It was shown to achieve higher rates of normoprolactinemia and to normalize PRL in BRC resistant patients [12–14]. Lastly, CAB is administered once or twice weekly compared with daily dose of BRC [9, 10] rendering BRC less convenient for most patients and possibly decreasing their compliance.

#### Dopamine agonists - PRL response

Since the 1990s CAB was studied as the main therapeutic option for hyperprolactinemia in general [15] and macroprolactinoma in particular [16–29]. The efficacy of CAB in reducing or normalizing PRL in patients with macroprolactinomas has been described in many studies (summarized in Table 1) in which following treatment with CAB, normal PRL was achieved in 75–95 % of patients (mean, 79.6 %).

**Table 1:** PRL and adenoma size dynamics in patients harboring macroprolactinoma treated with CAB

Author (year)	n(M/F)	Mean PRL (ng/ml) 1st/current	PRL normalized n(%)	Shrinkage^b^ n(%)	Unique study characteristics
Ferrari C (1997) [29]	85 (29/56)	300^a^/NA	52/85 (61.2 %)	41/62 (66.1 %)	
Colao A (1997) [22]	23 (8/15)	841/12	19/23 (82.6 %)	14/23 (61 %)	Low dose CAB
Pontikides N (2000) [23]	12 (6/6)	700/7	12/12 (100 %)	12/12 (100 %)	CAB as 1st line therapy
Colao A (2004) [24]	41 (41/0)	2019/17	31/41 (75.6 %)	41/41 (100 %)	Outcome was semen analysis
De Rosa M (2006) [25]	32 (32/0)	2705/93	31/32 (96.8 %)	NA	Outcome was quality of seminal fluid
Raverot G (2009) [26]	28 (17/11)	NA/NA	27/28 (96.4 %)	27/28 (96.4 %)	Visual field dynamics on CAB
Ono M (2010) [20]	29 (0/29)	348/6	29/29 (100 %)	29/29 (100 %)	Outcome was fertility
Bhansali A (2010) [19]	15 (15/0)	6249/47	14/15 (93 %)	15/15 (100 %)	Rapid CAB dose escalation
Karavitaki N (2012) [21]	12 (11/1)	2452/NA	11/12 (91.6 %)	12/12 (100 %)	Recovery of hypopituitarism
Corsello SM (2003) [17]	10 (10/0)	5794/77	5/10 (50 %)	9/10 (90 %)	Giant prolactinomas
Shimon I (2007) [16]	12 (12/0)	14383/15	10/12 (83.3 %)	9/11 (81.8 %)	Giant prolactinomas
Cho EH (2009) [18]	10 (10/0)	11426/109	5/10 (50 %)	10/10 (100 %)	Invasive giant prolactinomas
Total	309 (191/118)	2493/38	246/309 (79.6 %)	219/253 (86.6 %)	

^a^Median; ^b^Criteria for significant shrinkage varied between studies

Table summarizes publications including ≥10 male subjects, with data on patients with CAB-treated macroprolactinomas

Cabergoline was shown to be superior over other DAs in several respects, including efficacy. Colao et al. [30] described a cohort of patients treated with CAB for macroprolactinomas, either as 1^st^ line, or after treatment with other DAs. Treatment with CAB achieved normal PRL levels in 80.8 % (21/26), 51.3 % (19/37) and 94.7 % (18/19) of patients naïve, resistant or intolerant to other DAs, respectively. In almost all patients, a reduction of at least 50 % from baseline PRL level was achieved [9].

In addition to its direct effect on PRL, treatment with CAB has been effective in improving semen analysis in men [25] and when used to treat hyperprolactinemic women with amenorrhea, was found superior to BRC in gonadal function restoration (72 % vs. 52 %, CAB vs. BRC, respectively) [15].

#### Dopamine agonists - adenoma shrinkage

Apart from PRL normalization and hormone secretion recovery, tumor shrinkage to relieve tumor mass effects and prevent neurological complications is another treatment goal for patients harboring macroprolactinomas [6]. Dopamine agonists decrease the size of macroprolactinomas in most patients [6, 31, 32]. In a study by Colao et al. [30], treatment with CAB achieved adenoma disappearance in 61.5 % of patients naïve to DA treatment and an additional 30.8 % demonstrated adenoma shrinkage of >80 %. Patients intolerant or resistant to other DAs showed lower adenoma shrinkage rates (42.1 % and 30.3 %, respectively) [30]. First response to therapy may be expected as soon as a week or two after treatment initiation. However, in some patients shrinkage may become noticeable after only 6 months of therapy [31]. Most of the studies (8/11) verified adenoma shrinkage in response to CAB in all [18–21, 23, 24], or almost all [17, 26] patients, totaling 86.6 % of treated patients (see Table 1).

#### Dopamine agonists – resistance

Resistance to DAs has several different definitions in the literature, including failure to achieve normal PRL levels or adenoma shrinkage of >50 % [13, 33], failure to reduce PRL by >50 %, or to induce ovulation in women [33], or failure to reduce symptoms or normalize PRL despite CAB dose ≥2 mg/week [34]. Adenoma shrinkage is considered an inferior parameter for CAB resistance due to the limited data in the literature regarding the correlation with PRL control [13]. The prevalence of CAB resistance according to this criteria is 11 % among patients harboring macroprolactinomas [13]. Vroonen et al. [34], published a study of 92 patients with prolactinoma resistance to CAB, including 41 men with macroprolactinomas. In this study adenomas resistant to CAB defined a group of patients with a more advanced disease and potentially aggressive or even malignant tumors. In the minority of cases, other therapeutic options such as surgery or external radiation might be considered, as will be discussed later.

#### Dopamine agonists – safety and side effects

Rapid shrinkage of prolactinomas may cause pituitary apoplexy, which is discussed in detail below, see “Complications – Apoplexy”. Moreover, adenoma shrinkage might cause traction of the optic chiasm and secondary visual deterioration in patients with chiasmal damage [26, 35, 36]. Dopamine agonists are used for the treatment of Parkinson’s disease, at a daily dose of at least 2 mg, compared with a typical dose of 0.5–2 mg/week for macroprolactinomas. Patients treated for Parkinson’s are at moderate-to-severe risk for cardiac valvular damage [10]. However, several studies have shown that CAB treatment for prolactinomas was not associated with a clinically significant risk for valvular disease [37, 38]. Hence, the current guidelines do not recommend routine echocardiography for patients receiving a typical dose and suggest cardiac-echocardiographic surveillance only for patients treated with very high CAB doses for prolonged periods [10].

The most common adverse effects of DAs are gastrointestinal (nausea, constipation), dizziness (postural hypotension), headaches and nasal congestion [6]. Other less frequent side effects include fatigue, anxiety, cold sensitive vasospasm and psychosis. The side effect profile of CAB is better than BRC [6], making CAB preferable in routine practice. Leakage of cerebro-spinal fluid (CSF) following therapy with DA has also been described [39, 40], especially after rapid shrinkage of invasive adenomas [41].

#### Dopamine agonists – treatment duration

The treatment duration with DA for macroprolactinomas should be individualized, and depends on the tumor size and response to the treatment [6]. In one study [42], withdrawal from therapy according to strict criteria on follow-up (achieving normal PRL, disappearance or shrinkage of ≥50 % of tumor size, and a 5 mm distance from the optic chiasm) was associated with recurrence rate of 36 % 18 months following CAB cessation and a higher recurrence rate (53.1 %) after a longer follow-up (24–96 months) period [27]. In line with these results, guidelines for management of macroprolactinomas [9] suggest gradual decrease of CAB dose after two years of treatment, when PRL is normal and a shrinkage of ≥50 % is depicted. Among patients with macroprolactinomas, the presence of both low PRL levels and adenoma disappearance provided 67 % assurance for permanent or very long-standing remission, compared with only 22 % assurance for patients with a visible remnant on MRI [42].

### Other treatments

#### Surgery

Medical treatment with DAs is the preferred first-line treatment for macroprolactinomas [6, 9, 10], as it is highly effective in most patients (Table 1). However, there are several indications for pituitary surgery that might be applicable for a selected group of patients, specifically those who cannot tolerate, or are resistant to therapy with DAs, patients that seek fertility and harbor adenomas that impinge on the optic chiasm, psychiatric patients with contraindication to DA treatment and patients presenting with pituitary apoplexy or CSF leak [6]. Cystic prolactinoma is a unique entity, which usually does not shrink enough under DA treatment and surgery should be considered in invasive cases [6, 9, 10].

The preferred surgical approach is a trans-sphenoidal operation, whereas the trans-cranial approach is reserved for large inaccessible tumors. The success rates depend on the experience of the neurosurgeon [9], but even with experienced surgeons, the chance for persistent hyperprolactinemia may be high [43–46]. The expected success rate for normalizing PRL post-operatively is 65–85 % for microprolactinomas but less than 40 % for macroprolactinomas [47]. Furthermore, the long-term cure rate, defined by normal PRL levels, was only 16 % for macroprolactinomas [47]. Thus, pituitary surgery for macroprolactinomas usually results in significant tumor debulking, but without PRL normalization.

#### Radiotherapy

Radiotherapy is rarely used for PRL-secreting tumors. Radiotherapy confers several significant adverse effects, including vascular damage and increased future risk for stroke, hypopituitarism, damage to the optic chiasm and secondary brain tumors [9]. Radiotherapy is advised in cases of DA resistance, malignant prolactinoma and unsuccessful surgery [48, 49].

There are several radiotherapy techniques available. Fractionated stereotactic radiotherapy is saved for large lesions and requires 25–30 divided doses [50]. By contrast, radiosurgery techniques (e.g., gamma knife, cyber knife, proton beam) might facilitate the required radiation delivery in a single dose. Radiosurgery achieved biochemical remission in 27 % after 36 months and demonstrated improvement in 54 % of patients with invasive prolactinomas [51]. In comparison, gamma knife radiosurgery achieved a remission rate in 42 % of patients with Cushing’s disease, 22 months following radiation [52] and in 50 % of patients with growth hormone secreting pituitary adenomas after 36 months [53].

### Complications

#### Mass effect

The growing pituitary mass may impinge the surrounding structures, depending on the direction and severity of the extension. Organs that might be harmed include the optic chiasm, the cranial nerves located in the cavernous sinuses (namely the optic, trochlear, abducens and two branches of the trigeminal nerves) and other adjacent structures, such as the temporal lobe, the nasal cavity and sinuses, the internal ear and the thalamus. The related symptoms are mainly headaches and neuro-ophthalmological, including visual field alterations and ophthalmoplegia. Giant prolactinomas might also affect remote tissues (see paragraph entitled “Giant Prolactinomas”).

Visual field defects occur more often in larger adenomas [6] and necessitate the evaluation of visual fields in lesions abutting the optic chiasm [9]. The treatment of choice for tumors causing visual field defects is DA and recovery is expected in 75 % of patients, with early effects a few weeks following treatment initiation [30]. In our summary of the data of 72 patients with macroprolactinomas and visual field defects treated with DAs, 83 % (60/72) improved their vision following medical treatment (Table 2).

**Table 2:** Effect of medical therapy on visual field defects in patients with macroprolactinomas

Author (year)	n(M/F)	VFD n(%)	VFD improved n(%)	Treatment	Unique study characteristics
Ferrari CI (1997) [29]	85 (29/56)	12/85 (14 %)	6/12 (50 %)	CAB	
Colao A (1997) [22]	23 (8/15)	10/23 (43 %)	9/10 (90 %)	CAB	Low dose CAB
Pinzone JJ (2000) [101]	34 (34/0)	14/19 (74 %)	11/14 (79 %)	DA	Primary medical therapy
Pontikides N (2000) [23]	12 (6/6)	4/12 (33 %)	3/4 (75 %)	CAB	CAB as 1st line therapy
Sibal L (2002) [35]	35 (35/0)	18/35 (51 %)	18/18 (100 %)^a^	DA	Medical therapy
Corsello SM (2003) [17]	10 (10/0)	7/10 (70 %)	6/7 (86 %)	CAB	CAB for giant prolactinomas
Shimon I (2007) [16]	12 (12/0)	7/12 (58 %)	7/7 (100 %)	CAB	Giant prolactinomas
Total	211 (134/77)	72/196 (37 %)	60/72 (83 %)		

*DA* Any dopamine agonist, *CAB* Cabergoline only

^a^Four patients had secondary visual field deterioration due to optic chiasmal traction, after primary improvement

#### Hypopituitarism

Several studies evaluated the rates of pituitary axes dysfunction in patients harboring macroprolactinomas and the recovery rates of these deficits following treatment [30, 35, 54, 55]. The gonadotroph axis is most often damaged (73–86 %), presumably due to the double effect of the macroprolactinoma on this axis: increased pressure on the gonadotroph cells from the expanding mass and suppression of GnRH secretion by PRL effect in the hypothalamus [7]. Nevertheless, as has been previously shown [56], normal testosterone levels do not exclude the presence of PRL-secreting adenoma. Central hypothyroidism and hypocortisolism might also be induced by macroprolactinomas, though less often (18–41 % and 12–23 %, respectively) [30, 35, 54, 55]. Somatotroph axis evaluation is limited in patients with PRL-secreting tumors, due to the possibility of GH and PRL co-secretion in 10 % of these adenomas.

Recovery of the gonadotroph axis was reported in most patients with macroprolactinomas [21, 30, 54, 55]. The data regarding other pituitary hormones is less consistent. We found some recovery of the corticotrophs with no thyrotroph recovery [55], Colao et al. [30], found similar patterns, Karavitaki et al. [21] showed recovery of thyrotrophs in 25 % of affected patients but no recovery of ACTH secretion and Sibal et al. [35] demonstrated re-secretion of both ACTH and TSH in some patients in their cohort.

#### Cerebrospinal fluid leak

A leak in the CSF is usually iatrogenic, due to surgery or aggressive DA treatment, although it might be the presenting symptom in some macroprolactinomas [17, 19, 57, 58]. In a recent report on this complication of various pituitary adenomas [59], PRL-secreting tumors were reported in 81 % (42/52) of cases, many of these being giant prolactinomas. Meningitis, a complication of CSF exposed to the outer environment, was reported in 15–20 % of cases [60].

#### Apoplexy

Pituitary apoplexy is characterized by a rapid enlargement of the pituitary due to hemorrhage or infarct [61]. Although this is an uncommon complication, it is potentially life threatening, characterized by severe and abrupt headache, together with nausea, vertigo and meningismus [62]. Other symptoms might include acute hypopituitarism and neurologic compromise, including deteriorated consciousness, ophthalmoplegia and restriction of visual fields. Although the syndrome is usually acute and obvious, it may be subtle or even clinically silent [62].

Among pituitary tumors, apoplexy tends to occur in larger lesions, due to increased discrepancy between the rate of neoplastic progression and blood supply [62]. Lubina et al. [63] reported a group of 40 patients presenting with pituitary apoplexy, of them 63 % harbored non-secreting adenomas (NFPAs) and 31 % - prolactinomas. Semple et al. [61] showed that 77 % of patients with pituitary apoplexy (48/62) had pathologically confirmed NFPAs, while only one had prolactinoma [61]. Importantly, not only treatment with DAs might cause apoplexy, but also its withdrawal [64], possibly due to rapid re-growth of the adenoma [65].

The management of pituitary apoplexy depends on the clinical manifestations and their severity. A main consequence of apoplexy is the adrenal crisis. Thus, administration of hydrocortisone is indicated immediately on diagnosis, in addition to appropriate glucocorticoid coverage afterwards. Transsphenoidal surgery for decompression of the sella is indicated in patients with significant visual compromise or with a diminished level of consciousness [9, 62], whereas conservative management is optional for others. Furthermore, patients with apoplexy secondary to prolactinomas, without indications for surgery, were treated with DA with a good clinical outcome [63].

### Special populations

#### Postmenopausal women

Women usually present with microprolactinomas, in the age range of 20–40 years, whereas men more frequently present with macroprolactinomas [66, 67]. Interestingly, postmenopausal women tend to present with larger and more invasive tumors compared with premenopausal patients [68]. The classical explanation for this phenomenon is the early recognition of hyperprolactinemia due to menstrual disturbances. However, others have suggested that the low estrogen milieu might be associated with greater growth potential [3] and increased mitotic activity [2], though this notion is still controversial.

Shimon et al. [68] described a group of 14 postmenopausal women with prolactinomas. Thirteen (93 %) harbored macroprolactinomas, 4 of them giants (maximal diameter, 38–50 mm). Among patients treated with CAB, 83 % achieved normal PRL levels, 2–6 months following treatment initiation. The symptoms of six patients presenting with visual disturbances or diplopia were resolved following medical treatment. The authors concluded that despite the large and invasive tumors included in this unique group, they responded well to DA treatment.

#### Elderly

Macroprolactinomas might present at any age, though its prevalence decreases in the elderly [1, 3]. The prevalence of macroprolactinomas is higher in men, albeit they tend to present as macroadenomas both among older men and women [3, 68, 69]. There are several challenges in the diagnosis of such patients. First, symptoms of hypopituitarism in general and hypogonadism in particular are less frequent in older patients with prolactinomas [70] and might be mistakenly attributed to other comorbidities, which are common in this age group [71]. Second, visual disturbances might also remain undetected [70] or masked by other eye pathologies [71], such as cataract. Dopamine agonists are the mainstay of treatment and are usually effective in the elderly [70], with a lower incidence of DA resistance compared to younger patients. Nevertheless, in the selected cases requiring surgery, this latter option is safe in the elderly, as shown for NFPAs [72, 73].

#### Children

Prolactinoma is the most prevalent pituitary adenoma in children, representing half of these patients [74]. Nevertheless, prolactinomas are rare among children, although other pituitary adenomas in this age group were comparable to adults [75]. Most children harboring PRL-secreting tumors have macroadenomas [74], which might explain their tendency to develop neurologic complications. The presenting symptoms are usually delayed puberty and in girls primary amenorrhea and galactorrhea [9, 76]. Prolactin-secreting adenomas were reported in the context of the Familial Isolated Pituitary Adenoma (FIPA) syndrome linked to *AIP* gene mutation, although it is usually associated with acromegaly [77–79]. Screening for the Multiple Endocrine Neoplasia type 1 (MEN1) syndrome in the mutations of the *menin* gene might also be possible [80]. Salenave et al. [76] found mutations in *AIP* and *menin* in 9 % and 5 %, respectively, in children with macroprolactinomas. In this cohort, 74 % of patients (56/76) normalized PRL following DA treatment and tumor shrinkage was achieved in 76 % (56/74) of patients, with a median CAB dose of 1.5 mg/week. Seventeen children in this group required surgery and 2 patients underwent radiotherapy.

#### Pregnancy

Pregnant women with macroprolactinomas constitute a unique challenge to the clinician. Diagnostically, PRL gradually increases throughout pregnancy and cannot be used for the estimation of tumor size dynamics. Nevertheless, 30 % of macroadenomas grow during pregnancy [6, 81]. This is attributable to lactotroph hyperplasia in the estrogen milieu developed during pregnancy. Routine MRI follow-up during pregnancy is not indicated for intra-sellar adenomas without clinical signs of tumor growth [9, 10]. However, severe headache and/or visual disturbances should prompt formal visual field assessment and MRI, if needed.

Therapeutically, since DAs cross the placenta, treatment for hyperprolactinemia should be avoided during pregnancy if possible [9, 10]. However, this is difficult to implement, since pregnancy is usually diagnosed at week 5 (with delayed menstruation), by then the critical period of organogenesis has already started [82]. The safety of BRC during pregnancy was confirmed widely [83, 84], as was for CAB, though to a lesser extent [81, 85–91]. Thus, in case of a large adenoma or symptomatic growth during pregnancy, treatment with BRC should be initiated [10]. In the rare case of a woman who is planning pregnancy and who has a resistant macroprolactinoma or has intolerability to DA, pituitary surgery before pregnancy should be considered [10].

#### Giant prolactinomas

Giant prolactinomas are defined as adenomas larger than 40 mm [57]. Although these tumors consist only 2–3 % of all prolactinomas, they present some therapeutic challenges and unique complications. Giant prolactinomas may invade relatively remote structures and present with clinical manifestations including epistaxis, proptosis, nasal obstruction, hydrocephalus, tinnitus, hearing deficits and temporal epilepsy [48, 57].

A possible pitfall in the diagnosis of giant prolactinomas is the “high dose hook effect”. When measuring PRL levels using a two-site immunometric method, such as immunoradiometric assay (IRMA) or chemiluminescent immunoassay (ICMA), a very high PRL concentration might saturate the binding sites, causing falsely low PRL results. The practical solution when evaluating large pituitary masses, is to repeat the measurement of PRL levels after dilution of the blood sample [92].

Maiter et al. [57] published a recent review on giant prolactinomas. Prolactin response to DA (defined as PRL <25 ng/ml) was achieved in 60 % (58/97) of the patients in their cohort. Maiter also showed that medical treatment with DAs achieved significant tumor shrinkage (>50 %) in most patients, though no reliable pretreatment predictor for shrinkage was identified [57]. In other reports, DAs were also very effective in achieving prompt relief of hydrocephalus caused by giant prolactinomas [93, 94]. It is important to avoid treatment initiation with high DA doses, thus to decrease the probability for complications, such as CSF leak and tumor apoplexy [95].

Surgery is a second line option for the treatment of giant prolactinomas, as it confers morbidity risks [67] and usually fails to normalize PRL or to remove the entire tumor [96, 97]. From 97 patients with giant prolactinomas, only 14 required operation [57]. The indications for surgery were intolerance to DA, CSF rhinorrhea and resistance to DA with insufficient response or tumor progression [57].

#### Malignant prolactinomas

Malignant pituitary tumors are rare, with prolactinomas constituting about third [98]. The initial presentation of malignant prolactinomas may be identical to benign invasive macroprolactinomas [99]. However, the development of DA resistance in a compliant patient or increase in PRL in discrepancy with stable sellar mass, should serve as a red alert for malignancy or distant metastases and prompt further investigation including pituitary biopsy. Metastases of malignant prolactinomas involve most commonly the central nervous system, but 40 % may show systemic spread to bones, lymph nodes, lungs and ovaries [98].

The management of malignant prolactinomas consists of medical therapy, surgery, radiotherapy and chemotherapy. Dopamine agonists should be used in the highest tolerable dose to control the hyperprolactinemia [99]. Surgical management should be individualized, and surgical debulking can be performed as palliation, but is rarely curative [99]. Radiotherapy can be employed in either fractionated or radiosurgery techniques, depending on the spread and location of the lesions (see above “Other treatments – Radiotherapy”). Temozolomide is an alkylating agent used for pituitary carcinomas, which depletes the DNA repairing enzyme o-methyl guanine DNA methyltransferase (MGMT), but has shown limited biochemical or tumoral effect in patients with malignant prolactinomas [100]. The prognosis of PRL-secreting carcinomas is poor, with only 60 % of patients surviving more than 1 year [98].

## Conclusions

Prolactin-secreting macroadenomas are more common among men and older patients. Although they commonly present as large and invasive tumors, they usually respond to treatment with DAs and treatment in most cases is well tolerated. However, there are unique characteristics in different patient populations that might affect clinical therapeutic decisions and prognosis.

## Abbreviations

ACTH: Adrenocorticotropic hormone
BRC: Bromocriptine
CAB: Cabergoline
CSF: Cerebrospinal fluid
DA: Dopamine agonist
DNA: Deoxyribonucleic acid
GH: Growth hormone
GnRH: Gonadothropin releasing hormone
MGMT: o-methyl guanine DNA methyltransferase
NFPA: Non-functioning pituitary adenoma
PRL: Prolactin
TSH: Thyroid stimulating hormone
